# Rapid Scotch Whisky Analysis and Authentication using Desorption Atmospheric Pressure Chemical Ionisation Mass Spectrometry

**DOI:** 10.1038/s41598-019-44456-0

**Published:** 2019-05-29

**Authors:** Barry L. Smith, David M. Hughes, Abraham K. Badu-Tawiah, Rebecca Eccles, Ian Goodall, Simon Maher

**Affiliations:** 1 Department of Electrical Engineering & Electronics, University of Liverpool, Liverpool, UK; 2 Department of Biostatistics, University of Liverpool, Liverpool, UK; 3 Department of Chemistry & Biochemistry, Ohio State University, Columbus, OH USA; 4 The Scotch Whisky Research Institute, The Robertson Trust Building, Edinburgh, UK

**Keywords:** Mass spectrometry, Mass spectrometry, Analytical biochemistry

## Abstract

Whisky, as a high value product, is often adulterated, with adverse economic effects for both producers and consumers as well as potential public health impacts. Here we report the use of DAPCI-MS to analyse and chemically profile both genuine and counterfeit whisky samples employing a novel ‘direct from the bottle’ methodology with zero sample pre-treatment, zero solvent requirement and almost no sample usage. 25 samples have been analysed from a collection of blended Scotch whisky (n = 15) and known counterfeit whisky products (n = 10). Principal component analysis has been applied to dimensionally reduce the data and discriminate between sample groups. Additional chemometric modelling, a partial least squares regression, has correctly classified samples with 92% success rate. DAPCI-MS shows promise for simple, fast and accurate counterfeit detection with potential for generic aroma profiling and process quality monitoring applications.

## Introduction

Scotch Whisky is a key UK export valued at £4.3 bn in 2017. In terms of sales volumes, two out of every three bottles of Scotch exported are blended Scotch or blended malts^1^. Blended Scotch Whiskies are crafted by amalgamation of at least one single malt and one single grain whisky, overseen by highly experienced blenders. Sensory analysis is used to determine which grain and malt Whiskies are selected to produce a consistently blended Scotch that must pass the test of the typically astute Scotch consumer. Due to its high value and premium status, there is a clear incentive for fraud and adulteration of this beverage^2^.

The counterfeiting of spirits can be divided into two types. There is the fraudulent imitation of legitimately branded products, including refilling, fabrication and tampering (brand counterfeits). There is also the marketing of products as a particular spirit type, such as Scotch Whisky, when they are not compliant with the required production and labelling legislation (generic counterfeits). Spirit brand owners and regulatory enforcement officers are amongst the stakeholders that contend with counterfeiting. Counterfeiting is unregulated and exhibits large variability in base ingredients, distillation and production processes^3^.

Detection and classification of counterfeit Scotch is important to the Scotch Whisky industry to maintain brand integrity, consumer confidence and ultimately profitability. Variability in counterfeit production has very serious implications for quality control with potentially life threatening consequences for consumers^4^. The Scotch Whisky industry obtains authenticity enforcement in law via the Scotch Whisky Regulations 2009, SI 2009/2890 governing the distillation and maturation of genuine whisky. These include: (i) distillation exclusively in Scotland, using water and malted barley to which other whole grain cereals can be added, (ii) aged entirely in oak barrels for no fewer than 3 years, (iii) free from additives with the exception of caramel colourant and water, (iv) minimum alcoholic strength of 40% by volume^5^.

Mass spectrometry (MS) methods are well established and reliable at detecting counterfeit Scotch. Authenticity is determined via quantification of volatile higher alcohol congeners and age-related congeners present in whisky^6,7^. Deviation from defined concentration limits for one or more analytes results in a suspect or counterfeit classification. For this purpose, high performance laboratory-based instrumentation and hyphenated techniques (GC-MS or HPLC-MS) are routinely used^8,9^. Relatively complex sample pre-treatment stages^9^ and long analysis times severely limit throughput rates. Some classification methods additionally require high resolution MS^10,11^. Increasingly sophisticated techniques and higher instances of counterfeiting motivates development of a high throughput solution that provides rapid assessment of authenticity. Developing such an analytical method to screen for counterfeit Scotch in a general-purpose fashion, must overcome the challenge of identifying counterfeits from a chemically diverse range of genuine samples.

Ambient ionisation (AI) MS is one the most promising routes toward high throughput MS-based screening for food safety^12–14^. AI approaches have certain advantages which generally lead to a reduction in experimental complexity with vastly reduced sample preparation, resulting in substantial improvements for sample throughput. AI MS has found increasing applications in food fraud detection, for example, direct analysis in real time (DART), a popular ‘point-and-shoot’ ambient ionisation source was used to analyse 343 red and white wine samples using metabolic fingerprinting and advanced chemometrics^15,16^. Recently, an AI technique known as paper spray^17–20^, that generates ions from a wetted paper substrate with a high voltage applied, has been used to classify whisky^21,22^.

Desorption atmospheric pressure chemical ionisation (DAPCI) is one of a number of AI sources capable of desorbing and ionising analyte molecules for mass spectrometric analysis directly from a sample in the open environment^23^. For example, DAPCI has been used to differentiate *Cinnamomum camphora* chemotypes directly from raw plant tissue^24^ and for the detection of melamine in powered or liquid milk^25^. DAPCI-MS offers some advantages over other AI techniques that make it especially suitable for direct, rapid chemical analysis of Scotch. As has been reported^26^, flavour components in whisky tend to co-localise preferentially at the liquid-air interface in mixtures that contain up to 45 vol-% of ethanol rather than in the bulk liquid. Low charged reagent gas flow rates enable direct liquid interrogation at precisely the site where the concentration of flavour components is maximised. We hypothesised that this can be accomplished by gently perturbing the surface of the whisky, direct from the bottle without back scattering of liquid droplets. DAPCI generates highly fluent primary charge carriers without the necessity for additional solvent and therefore avoidance of any liquid phase spray effluent. This makes DAPCI an ideal candidate for online process monitoring of analytical Scotch ‘fingerprints’ with batch variability compliance, where contamination from solvent spray effluent would be highly undesirable^27^. In this configuration, DAPCI requires virtually no sample usage and no sample preparation, which is highly advantageous for non-targeted approaches were sample preparation can impact on the accuracy of the analytical results^28^. Chen *et al*. have demonstrated that rapid collection of DAPCI-MS data can be reliably classified using chemometric techniques^29–31^. The DAPCI source has been successfully miniaturised to the point of hand-held portability^32,33^ and coupled directly to portable MS instrumentation^34^, demonstrating its feasibility for use in mobile environments. This opens a number of possibilities, for example, providing an on-the-spot assessment to provide sufficient grounds to seize and hold Scotch pending more thorough analysis.

**Figure 1:**
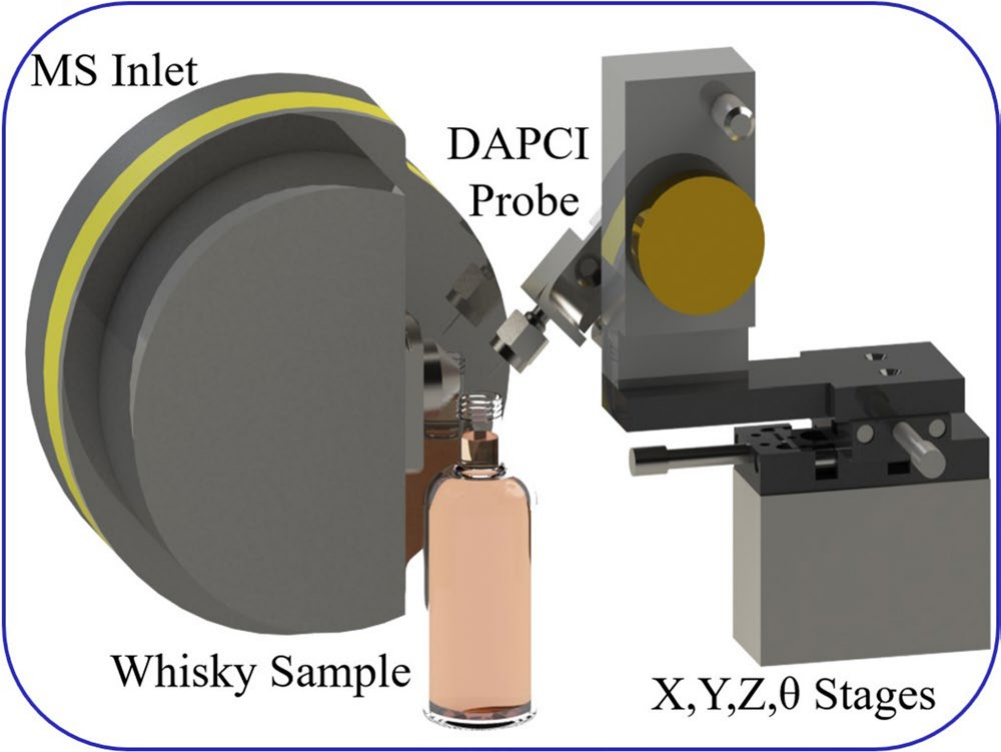
CAD illustration of DAPCI-MS analysis of Scotch.

In this study, DAPCI-MS was employed to collect mass spectra from a total of 25 whisky samples, without any sample preparation or solvent addition, direct from the bottle with virtually no sample usage; the entire sampling and analysis time is ~20 seconds per sample (Fig. 1). MS spectral ‘fingerprints’ were analysed using principal component analysis (PCA) and score plots obtained to visualise the difference between authentic and counterfeit whisky. Further modelling through partial least squares-discriminant analysis (PLS-DA) is used to predict authenticity of a test set from a training dataset. To the authors’ knowledge, this study represents the first time that direct-from-the-bottle chemical analysis and authenticity assessment of Scotch has been performed with an ambient desorption/ionisation source, providing rapid analysis without any sample preparation requirements.

## Results and Discussion

### Fingerprinting of authentic and counterfeit whisky samples by DAPCI-MS

To maintain signal stability for a relatively wide mass window, the average relative standard deviation (RSD) across all data points for the 10 measurements was deemed an appropriate metric to determine the optimum experimental conditions, as discussed in the methods section. The average RSDs for all mass points in each sample were as follows: 9.3%, 9.8%, 10.3% and 11.3% for brand 1, brand 2, brand 3 and counterfeits samples, respectively (Supplementary Fig. S1). A proportion of the variance can be accounted for by the batch production processes used when blending multiple whiskies into a consistently blended Scotch. The remaining variance is likely due to experimental fluctuations as a consequence of the inherent nature of direct sampling in the open environment. Given the simplicity of the experiment, reasonably high throughput rate (of 3 samples per minute) and lack of sample handling or pre-treatment stages, the variance within this experimental method is well-controlled.

**Figure 2:**
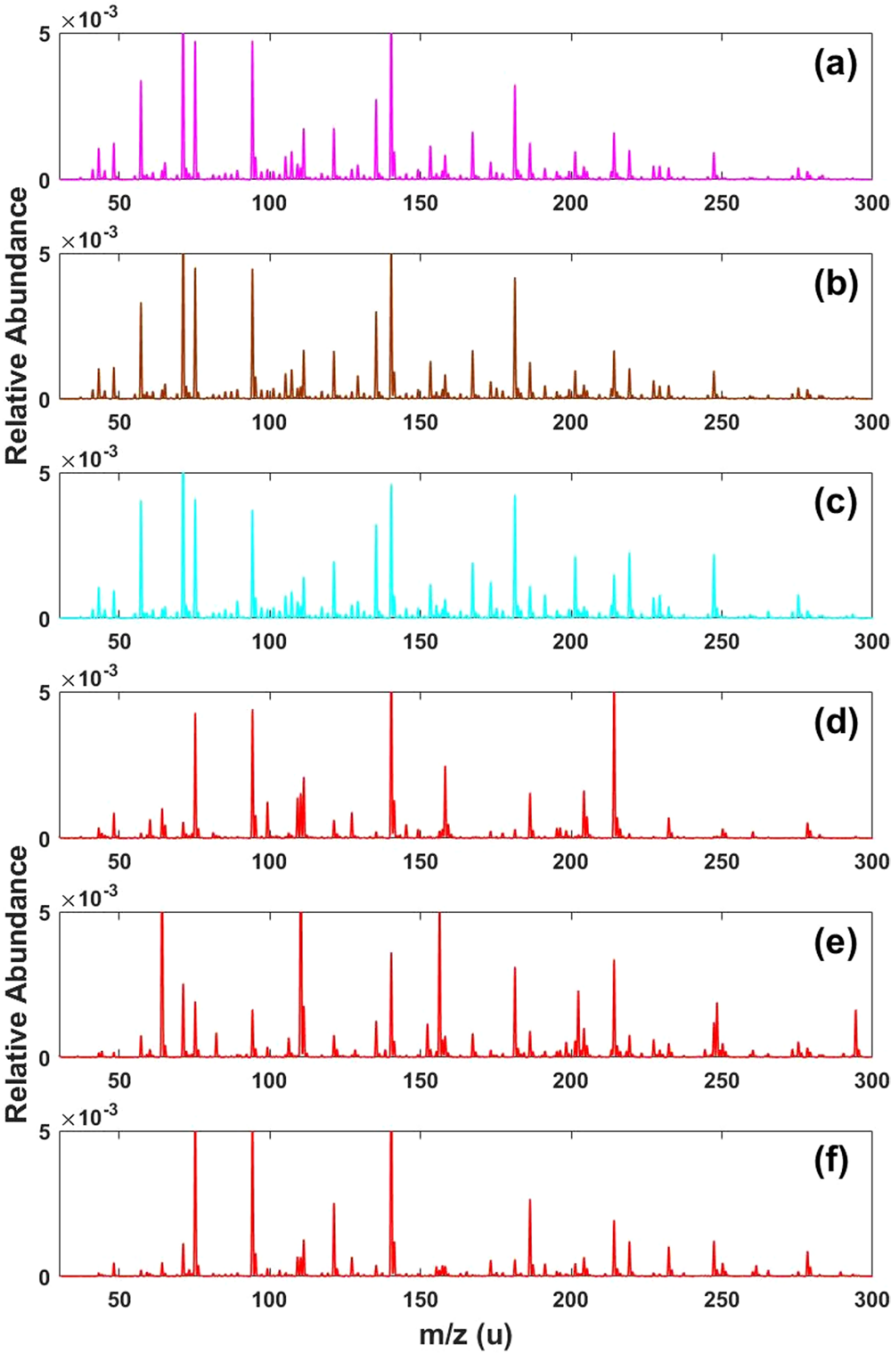
DAPCI-MS spectral ‘fingerprints’ from 3 authentic brands of whisky and 3 counterfeit samples (after post-processing to remove ethanol clusters). (**a**) Mean spectra of Brand 1. (**b**) Mean spectra of Brand 2. (**c**) Mean spectra of Brand 3. (**d**) Individual counterfeit sample S16-0226. (**e**) Individual counterfeit sample S16-0810. (**f**) Individual counterfeit sample S16-0940.

A rudimentary analysis of the dataset reveals distinct differences between authentic and counterfeit samples under DAPCI-MS interrogation. Averaged mass spectra from three branded Scotch whiskies and 3 counterfeits are shown in Fig. 2. Differences between the mass spectra for authentic and counterfeit samples can be readily observed by visual inspection. Moreover, the spectra from the three different brands show close similarity. A holistic consideration of the total number of peaks in the dataset, reveals an average increase of 7.6% in the authentic samples over counterfeit samples (Supplementary Fig. S2). This is not an unexpected result since it is well known that the maturation process in Scotch adds to the chemical richness of the whisky. Volatile aroma imparting compounds include lignin derived metabolites from the cask wood used to mature the Scotch^35^. Counterfeit samples will generally avoid (or skimp) the correct and lengthy (>3 years) maturation process in casks. Genuine samples are therefore more likely to show higher levels of chemical diversity reflecting the higher abundance of peaks present in the samples as a whole. The three counterfeit samples shown in Fig. 2((d) S16-0226, (e) S16-0810 and (f) S16-0940) exhibit distinct differences not only in respect to the genuine Scotch spectra but also amongst themselves. This is entirely expected since there is no typical counterfeit sample. The chemical composition of counterfeited Scotch naturally reflects the manufacturing processes used to defraud. Typical examples include: rebranding of lower quality less mature whiskies as being more expensive, mature whiskies; dilution of genuine produce with either water or ethanol; industrially produced ‘moonshine’, a term used to denote whisky that has not been through cask maturation; and, relabelling of foreign, mature whisky as Scotch. The latter is likely the most difficult to capture with DAPCI-MS. Further analysis, considering the total ion current distribution defined over a set of given *m*/*z* ranges, reveals further interesting trends. For instance, over the ranges of *m*/*z* 10–100, 100–200, 200–300 and 300–400, an average increase in the ion abundance of lower mass regions for authentic samples is observed; there is a corresponding increase in the higher mass regions for counterfeit samples (Supplementary Fig. S3). Such trends within the data could potentially be used to enhance classification.

### Chemical analysis of whisky samples by DAPCI-MS

All DAPCI spectra of Scotch are dominated by highly abundant protonated ethanol (M.W. 46) clusters in positive ion mode, *m/z* 47, 93, 139, 185, 231 and 277 (Supplementary Fig. S4). DAPCI operation in solventless mode utilises atmospheric water to facilitate primary proton donation to analyte molecules. In the case of whisky analysis, higher proton affinity (776.4 kJ/mol) and lower vapour pressure of ethanol in comparison to water (691 kJ/mol) causes water to be displaced as the primary reagent ion in favour of vapour phase ethanol. Due to the high abundance of ethanol in the native sample, it is not possible to de-solvate entirely the ethanol peaks. To visualise the spectral differences between samples, the highly abundant ethanol clusters were removed in software (Fig. 2). In Fig. 2, spectrum (a) is the mean for all brand 1 samples, spectrum (b) is the mean for all brand 2 samples and spectrum (c) is the mean of all brand 3 samples. The mean spectra for all three authentic brands exhibit remarkable consistency in terms of peak abundance and mass positions. A high degree of ‘chemical richness’ can be observed, reflecting the complexity and diversity of the native whisky matrix.

**Figure 3:**
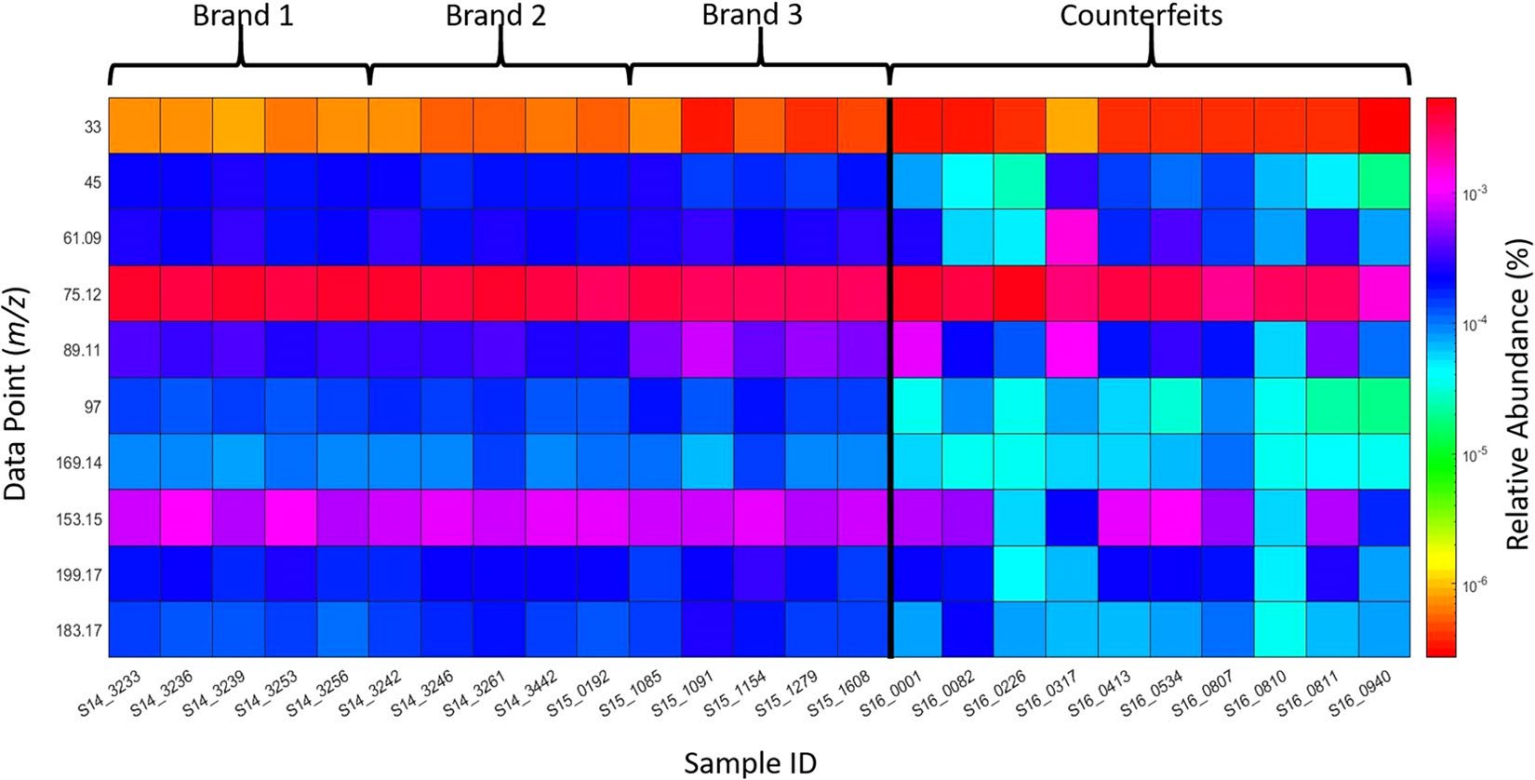
Intensity heat map for selected peaks m/z 33, 45, 61.1 75.1, 89.1, 97, 169.1, 199.2, 153.1 and 183.2 for each sample.

Well-known major constituents of whisky, aside from ethanol and water, are volatile alcohol congeners also called higher oils formed during ethanol fermentation. R. Aylott *et al*.^6^ devised a quantitative methodology to determine authentication of Scotch based on higher alcohol and related congener concentrations: Acetaldehyde (M.W. 44), Methanol (M.W. 32), Ethyl Acetate (M.W. 88.11), n-Propanol (M.W. 61.09), Iso-Butanol (M.W. 74.12), 2-Methyl-1-butanol (M.W. 88.15) and 3-Methyl-1-butanol (M.W. 88.15). Deviation from normal concentration ranges for one or more of the major congeners can be considered as a red flag for authenticity. Supplementary Fig. S4 shows the corresponding mass spectral peaks suspected to correspond to these alcohols in protonated form (*m/z* 33, 45, 61, 75.1, 89.1). A rapid qualitative assessment of authenticity can be obtained by observing the stability of genuine brands and significant deviation in at least two of the data points for the counterfeit samples. Further markers provided^6^ include age related congeners: Vanillic Acid (M.W. 168.14), Syringic Acid (M.W. 198.17), Vanillin (M.W. 152.15), Syringaldehyde (M.W. 182.17) and Furfural (M.W. 96). Figure 3 shows the relative abundance of the suspected corresponding protonated peaks (*m/z* 97 169.1, 199.2, 153.1 and 183.2), where significant deviation can be observed for all of the counterfeit samples for at least one of the marker peaks and eight of the counterfeit samples show deviation in at least two of the marker peaks.

### Principal component analysis of whisky by DAPCI-MS

**Figure 4:**
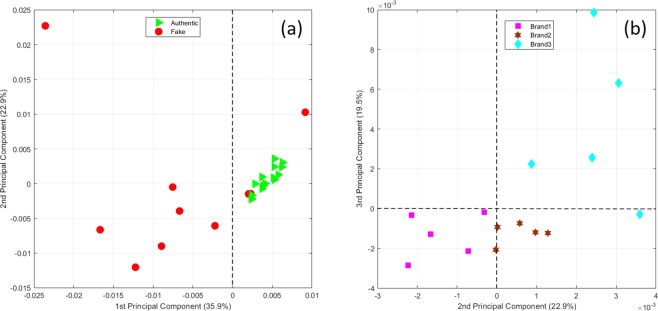
Principal component analysis results from DAPCI-MS analysis of Scotch: (**a**) PCA score plot of PC1 and PC2 with figure legend symbols denoting authentic (triangles) and fake (circles) samples. (**b**) PCA score plot of PC2 and PC3 with figure legend symbols denoting the different brands.

A principal component analysis (PCA) model was built using mean centred data on the first 3 principal components (PC) accounting for 78.3% of the total variance (35.9%, 22.9% and 19.5% for PC1, PC2 and PC3 respectively). PCA score plots are presented in Fig. 4(a) PC1 versus PC2 and (b) PC2 versus PC3. Examination of the PCA model shows good separation across the vertical axis on PC1, the component explaining the largest proportion of the variance. All of the authentic samples (*green triangles*) yield positive scores on PC1 and 70% of the counterfeit samples (*red circles*) yield negative scores (on PC1). Tightly clustered authentic samples and relatively dispersed counterfeit samples can be observed, reflecting the varied nature of counterfeiting and the similarity of the branded samples. Therefore, we can say that PC1 is the component that best characterises the authenticity of Scotch using DAPCI-MS. Examination of Fig. 3b, shows separation across both the 2^nd^ and 3^rd^ PCs based on the brand characteristics. PC2 appears a reliable indicator for brand characteristics: Brand 1 yields negative scores on the vertical axis, whilst Brands 2 and 3 give positive scores. Brand 3 can be separated along the horizontal PC3 axis, with four out of five samples yielding positive scores and all of brand 1 and 2 samples yielding negative scores. PC2 and PC3 scores are likely a function of the flavour profile, cask type, or maturation age of whisky.

**Figure 5:**
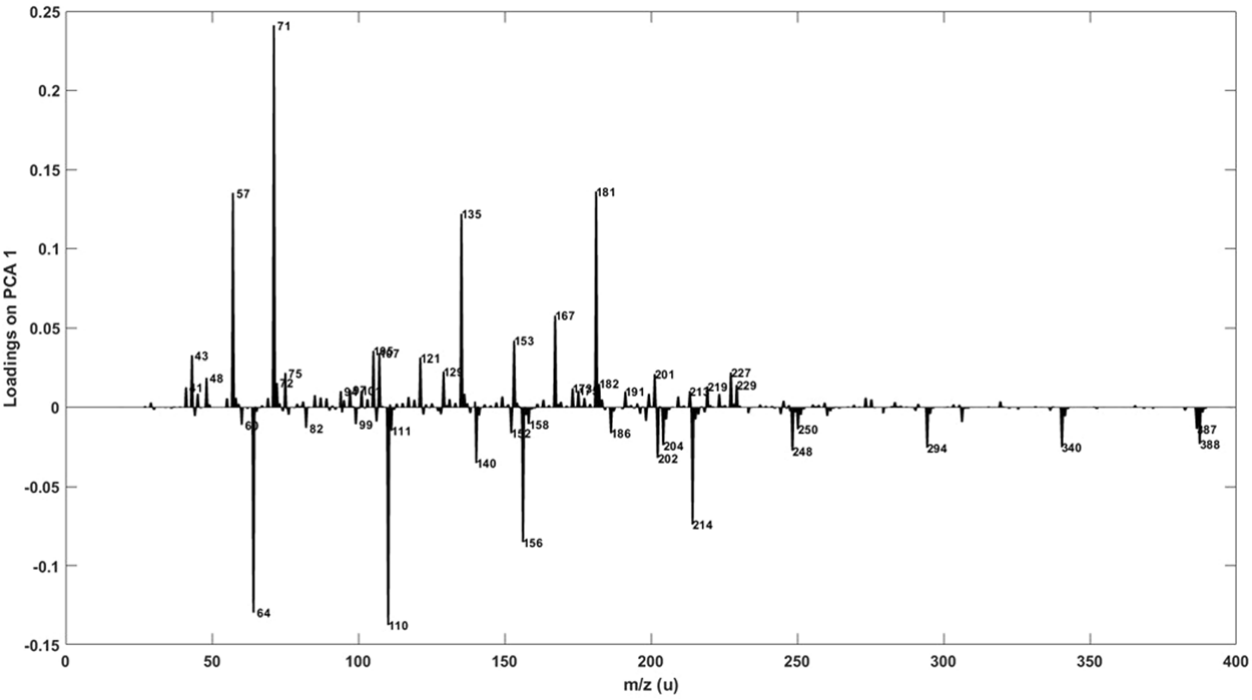
Principal component loading results for principal component 1.

Inspection of the loadings of PC1 (Fig. 5) indicates those variables most responsible for the separation of authentic and counterfeit samples. The largest contribution to authentic samples came from four markers *m/z* 57, 71, 135 and 181. Counterfeit samples could mainly be distinguished by four marker peaks at *m/z* 64, 110, 156 and 214. Supplementary Fig. S5 displays the relative intensity heat map for authenticity markers identified by the PCA loadings plot. It is clear that there is at least 1 marker ion in all of the counterfeit samples that significantly deviates in relative signal intensity from the genuine samples, whilst 7 counterfeit samples exhibit multiple deviations. The loadings plot for PC3 (Supplementary Fig. S6) indicates major markers for Brand 3 are cations *m/z* 173, 201, 219, 229 and 247. This can be readily observed by inspection of the mass spectra (see Fig. 2), where the peaks at *m/z* 201, 219 and 247 can be used to identify this brand. Interestingly, cation *m/z* 229.3 (which we confirmed as being ethyl dodecanoate (M.W. 228.3)) was recently identified as a major marker for authenticity using a chemical ionisation (CI) source in positive mode with high resolution MS instrumentation^11^. Ethyl dodecanoate is an ester commonly found in whisky which has a waxy aroma. In conjunction with other esters, it contributes to the primary aroma profile of whisky. In our case, the relative abundance at *m/z* 229.3 is consistently slightly lower in the counterfeit samples than the authentic samples (except in one case where it was significantly higher). Supplementary Fig. S7 confirms this identity, showing MS/MS data using collision induced dissociation (CID) for the parent ion, *m*/*z* 229.3 (from authentic Scotch sample S14-3233). The daughter ion at *m/z* 201, corresponding to a neutral loss of 28, is in agreement with Pichini *et al*.^36^ for the same cone voltage (20 V) and collisional energy (10 eV) who performed an LC-MS/MS experiment assessing the presence of alcohol in postnatal meconium.

An additional targeted PCA model (Supplementary Fig. S8) was generated using a smaller pool of suspected peaks assigned to alcohol congeners and age related congeners (*m/z* 33, 45, 75.1, 89.1, 97, 169.1, 199.2, 153.1 and 183.2), as illustrated in Fig. 3. All the of the authentic samples yielded positive PC1 scores and nine out of ten of the counterfeit samples yielded negative scores on PC1. Selection of features based on published methods to established Scotch authenticity, coupled with almost complete separation across PC1, suggests that the alcohol and age related congeners are desorbed and ionised during direct DAPCI-MS analysis of Scotch. This result suggests that DAPCI-MS has much potential to be used for targeted chemical assessments of Scotch and potentially other foodstuffs.

### Classification of genuine and authentic whisky by DAPCI-MS

**Figure 6:**
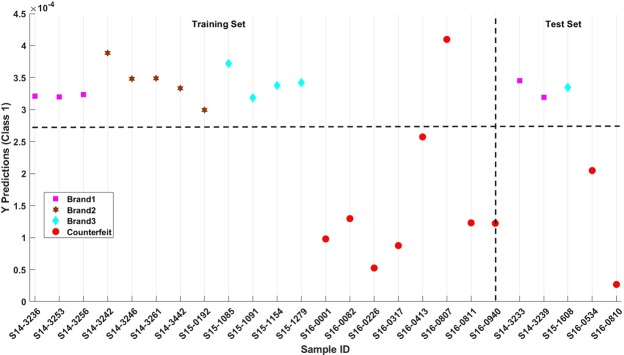
Predictions of the PLS-DA Model. Red circle markers denote counterfeit samples.

A partial least squares discriminant analysis (PLS-DA) model was compiled from the full dataset. Unlike PCA, PLS-DA is a supervised learning technique whereby class information is fed into the model rather than relying on visual inspection. Furthermore, PLS-DA enables class predictions of unknown samples based on model parameters generated from known sample data. The optimum PLS-DA model was built using only two latent variables (LV) accounting for 73.3% of the variance in *y* and 45.3% of the variance in *x*. Figure 6 illustrates an example of predicted values resulting from a PLS-DA model when randomly partitioning the data into training and 20% holdout test sets (datasets not used to generate the model). Only one of the training set samples was incorrectly classified (S16-0807). Prior testing by SWRI using conventional GC and LC methods found sample (S16-0807) to have a profile consistent with the higher alcohol and related congeners referred to earlier, and a profile of age related congeners which, whilst judged as inconsistent with acceptable cask maturation, was close to the limits of what might be considered acceptable. Model validation was performed as outlined in the methods section and the figures of merit for the model are as follows: sensitivity (true positive rate), 100%; specificity, 80% (true negative rate); area under receiver operating characteristic curve (AUROC), 0.89; and an overall classification error rate of 8%.

**Figure 7:**
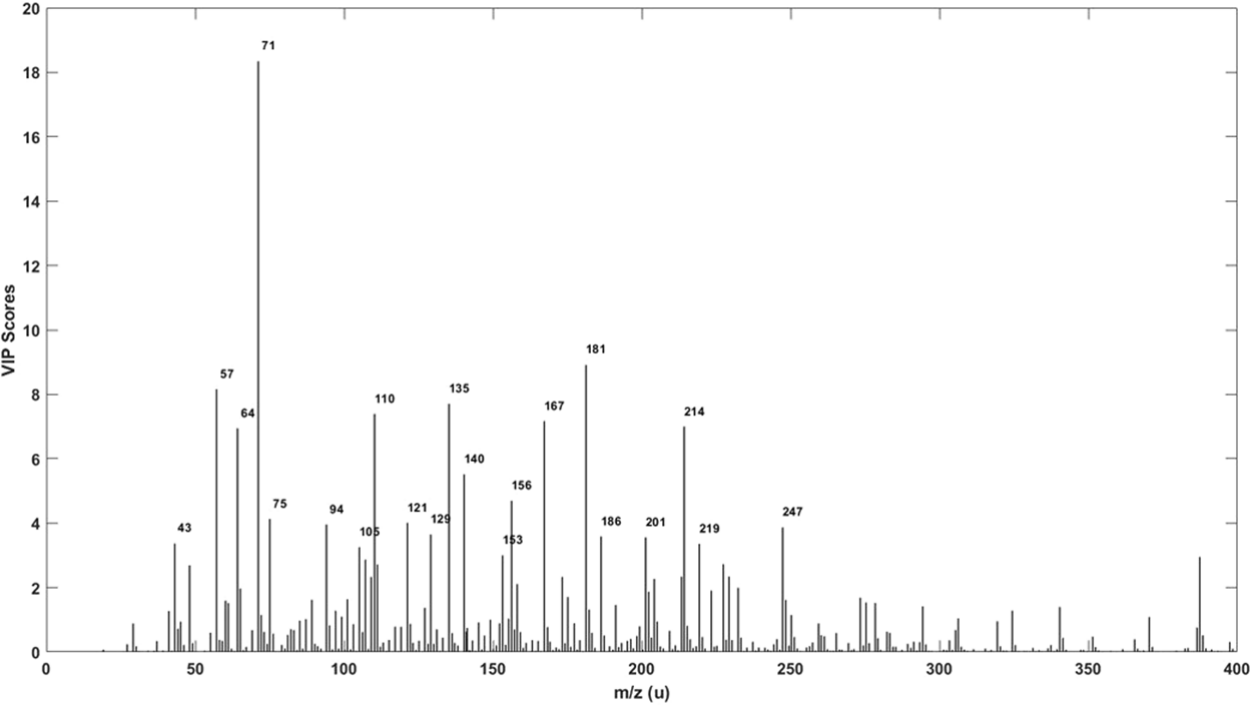
VIP score result from PLS-DA model.

Variables important in projection (VIP) score plot (Fig. 7), indicates the peak location and intensity values that contribute most to the class discrimination; the higher the score, the more important the variable. Figure 7 indicates that *m/z* 57, 71, 110, 135, 167, 181, 214 are the variables most responsible for the classification. It is in excellent agreement with the PC1 loadings plot (Fig. 4); the most important variables in each case are consistent. Almost identical results from two independently generated models is a good indicator of the reliability of the approach in Scotch authenticity classification using DAPCI-MS. Further improvements to the robustness, accuracy and generality of the model could likely be improved by increasing the sample set size.

## Conclusions

A non-targeted DAPCI-MS methodology combined with chemometric techniques, PCA and PLS-DA, has been utilised to determine blended Scotch Whisky authenticity. A sample set of 15 authentic branded samples (5x samples from 3 different Scotch brands, exhibiting batch variability) and 10 counterfeit samples were analysed directly from the bottle with no sample pre-treatment and virtually no sample usage, nor addition of any solvent. A successful classification rate of 92% was achieved for the samples analysed using PLS-DA. DAPCI-MS shows promising potential to be an effective technique for rapid *in-situ* screening to determine authenticity of Scotch. DAPCI is a ‘green’ ambient source requiring no additional solvents to generate clean, informative spectra. The performance of this approach coupled with the simplicity and speed of analysis opens up the possibility for continuous process monitoring to provide a qualitative assessment of batch analytical ‘fingerprints’. Despite the limited sample size, the high degree of separation in PCA space for authenticity and inter brand with batch variability is compelling evidence that the separation is based on (semi)volatile components found in Scotch and their production dependent profiles. It is reasonable to conclude that the methodology presented here would be of interest to producers of other high value spirits that are subjected to fraud such as wine, brandy and rum.

## Materials and Methods

### MS settings

All DAPCI-MS experiments were performed on a Waters Xevo triple quadrupole mass spectrometer (TQ MS). Full MS scan mode was used with a mass acceptance window of 10 to 400 amu. Each sample was analysed for 20 seconds (10 × 2 second scans) delivering a throughput rate of 3 samples per minute. The source temperature was maintained at 100 °C. The cone voltage was set to 50 V, optimised to attain good transmission of ions in the range of interest (10–400 amu) whilst ensuring a high degree of ethanol de-clustering. No further optimisations were performed and the remaining instrument settings were left as per the manufacturer recommendations.

### DAPCI-MS settings

**Figure 8:**
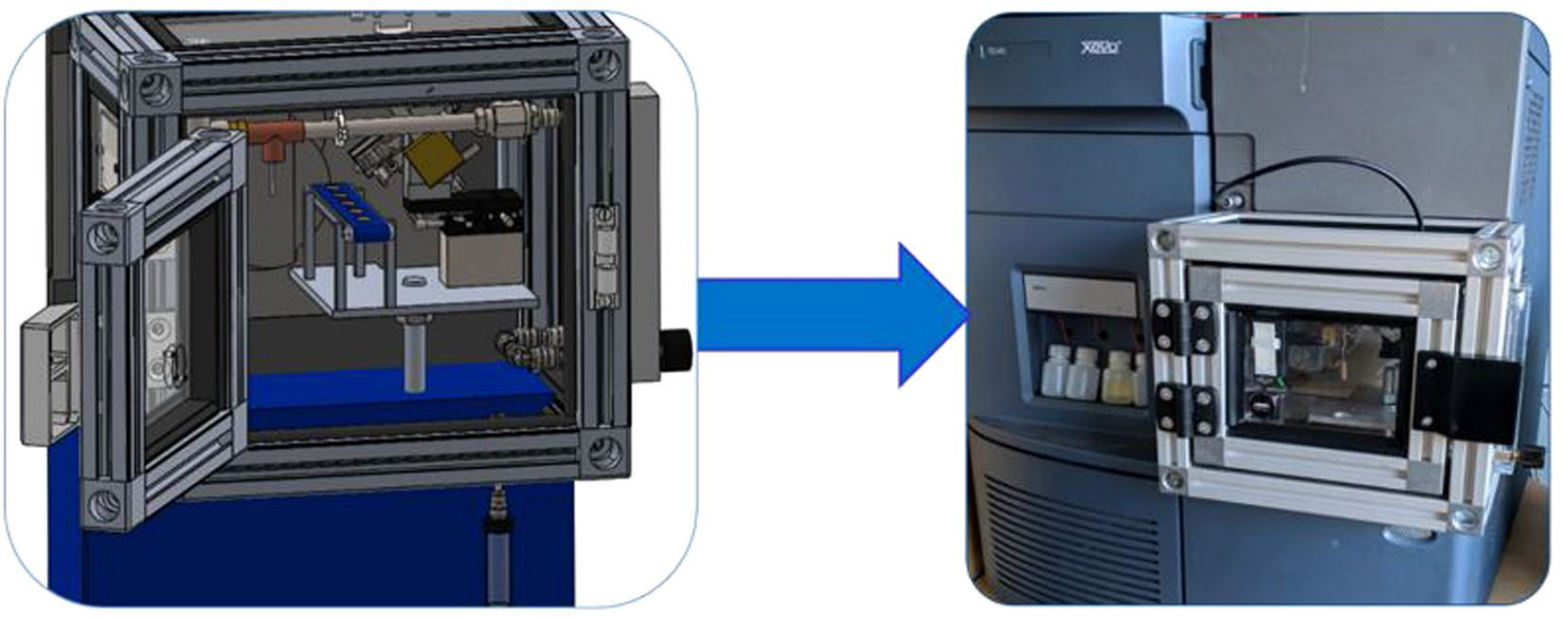
CAD illustration and implementation of the bespoke DAPCI-MS interface.

A bespoke DAPCI-MS interface was designed and constructed, to facilitate moderately high throughput, automated liquid sampling directly from a bottle and reliable control of experimental parameters. Figure 8 shows the design and construction of the interface. For this study, both MS and DAPCI were operated in positive ion mode. 4 kV was applied to the DAPCI electrode to facilitate a corona discharge. A nitrogen gas flow of 0.4LPM, controlled using a variable flow meter, was directed inline with the electrode as per normal DAPCI operation. A number of primary reagent ions and secondary cluster ions were generated to interact with the liquid surface, performing ionisation/desorption under ambient conditions. The nitrogen gas was further heated to 60 °C using an Omega Engineering inline heating element with integrated K-Type thermocouple providing feedback to an Omron E5CB PID temperature controller aiding thermal desorption of analyte ions within the complex whisky matrix. The DAPCI ion source was placed 8 mm from the inlet and at 30° to the sample. The distance between the needle tip and the sample was approximately 5 mm. Prior optimisation tests found that the probe to sample angle was of importance, in terms of the number of measured peaks and the measurement stability. Steeper angles >45° and higher gas flow rates, whereby the charged gas interaction with the liquid surface was more vigorous, intensified the number of peaks in the spectra, however this was accompanied by an increase in the overall RSDs. An angle of 30° and flow rate of 0.4LPM were selected as the gas was gently perturbing the surface which resulted in a reduction in the number of measured peaks but provided sufficiently stable data with acceptable variance. Angles lower than ~20° resulted in fewer peaks being present in the dataset.

### Chemicals and reagents

25 samples provided by the Scotch Whisky Research Institute (SWRI) comprising 15 genuine samples and 10 known counterfeits were analysed during this study. Authenticity and provenance were confidently determined prior to DAPCI-MS analysis using standard SWRI GC and LC methodologies. Supplementary Table S1 shows the labelling scheme employed within the study along with sample descriptions for each sample analysed. Samples were interrogated by DAPCI-MS in a random order to minimise any memory effects or residual carryover to reduce any bias to the subsequent data analysis. No additional solvent or pre-treatment stages were applied and the samples were directly analysed in the containers in which they arrived.

### Data analysis

Raw data collected from each sample was held in separate Mass Lynx files and given appropriate labels based on sample numbers found in Supplementary Table S1. Conversion from Mass Lynx.RAW to.mzXML file format was completed to aid post-processing and chemometric analysis in Matlab R2017b (Mathworks). The Bioinformatics toolbox function “mzxmlread”^37^ was used to extract the scan data in the mzXML file and produced a data structure containing the intensity information from the scan data held in each file. Each scan was normalised to the total ion current and smoothed using Matlab functions “msloss” and “msnorm”. Groups of 10 spectra were then averaged to produce 1 data row consisting of 6011 *m/z* points (independent variables) and associated relative intensity value (dependant variables). A final column assigning a categorical descriptor, authentic or fake, was appended to give a total data structure consisting of 25 rows and 6012 columns. Performance of the model was analysed following guidance from the work of Cavanna *et al*.^28^, 10 times leave-one-out cross-validation was used to assess model accuracy, and prevent overfitting.

## Supplementary information

Supplementary Information
