# Supplementary material for: Rapid Scotch Whisky Analysis and Authentication using Desorption Atmospheric Pressure Chemical Ionisation Mass Spectrometry
Source: Sci Rep. 2019 May 29;9:7994. doi: 10.1038/s41598-019-44456-0 (PMC6541643; doi:10.1038/s41598-019-44456-0)
Supplement: Supplementary file 1 — Supplementary Information [file 41598_2019_44456_MOESM1_ESM.pdf]

Supplementary Information

Rapid Scotch Whisky Analysis and Authentication using Desorption Atmospheric Pressure Chemical Ionisation Mass Spectrometry

Barry L. Smith[1], David M. Hughes[2], Abraham K. Badu-Tawiah[3], Rebecca Eccles[4], Ian Goodall[4] and Simon Maher*[1]

1) Department of Electrical Engineering & Electronics, University of Liverpool, Liverpool, UK;
2) Department of Biostatistics, University of Liverpool, Liverpool, UK;
3) Department of Chemistry & Biochemistry, Ohio State University, Columbus, OH, USA;
4) The Scotch Whisky Research Institute, The Robertson Trust Building, Edinburgh, UK;

*For correspondence, email s.maher@liverpool.ac.uk

Figure S1 depicts the average relative standard deviation (RSD) for each sample. This is the mean RSD of the signal intensity for every mass point in the 10 scans that comprises the dataset for each sample. The calculation was performed in Matlab by firstly computing the mean value of the signal intensity at each mass point in the 10 scans for each sample, after which the standard deviation is calculated for all mass points and then the RSD. Finally, the averaged RSD for each sample is taken as the mean of all of these RSDs (for each mass point within the 10 scans for a given sample). Each sample averaged RSD is shown in Figure S1. Descriptions for the sample IDs are listed in Table S1 (below).

From Figure S1 the mean RSDs for each brand and counterfeit samples can then be computed (Brand 1: samples 1-5, 9.3 %; Brand 2: samples 6-10, 9.8 %; Brand 3: samples 11-15, 10.3 %; Counterfeits: samples 16-25, 11.3 %).

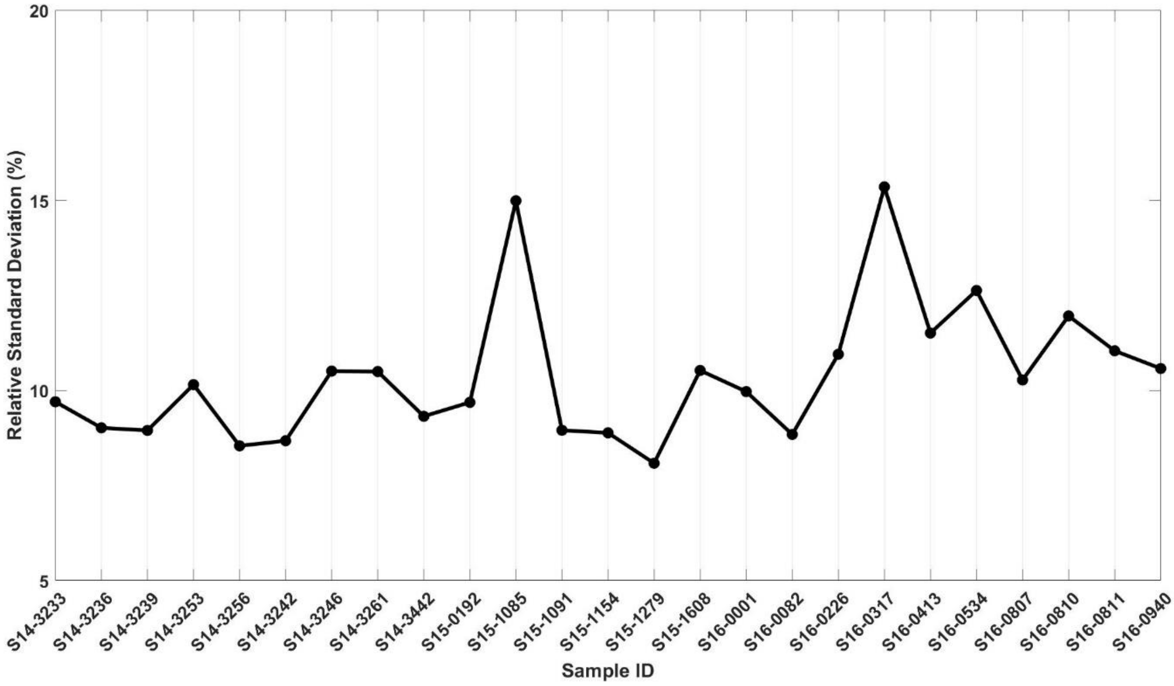

***Figure S1**: The average relative standard deviation across all data points for each sample analysed.*

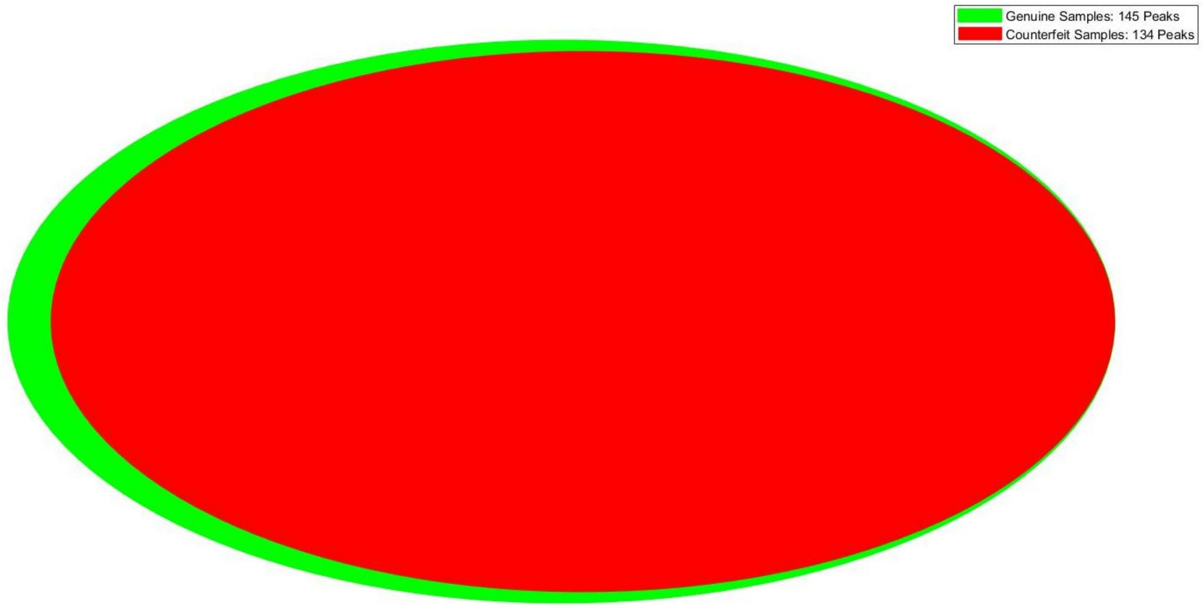

Figure S2: *The average number of individual peaks in the authentic and counterfeit datasets.*

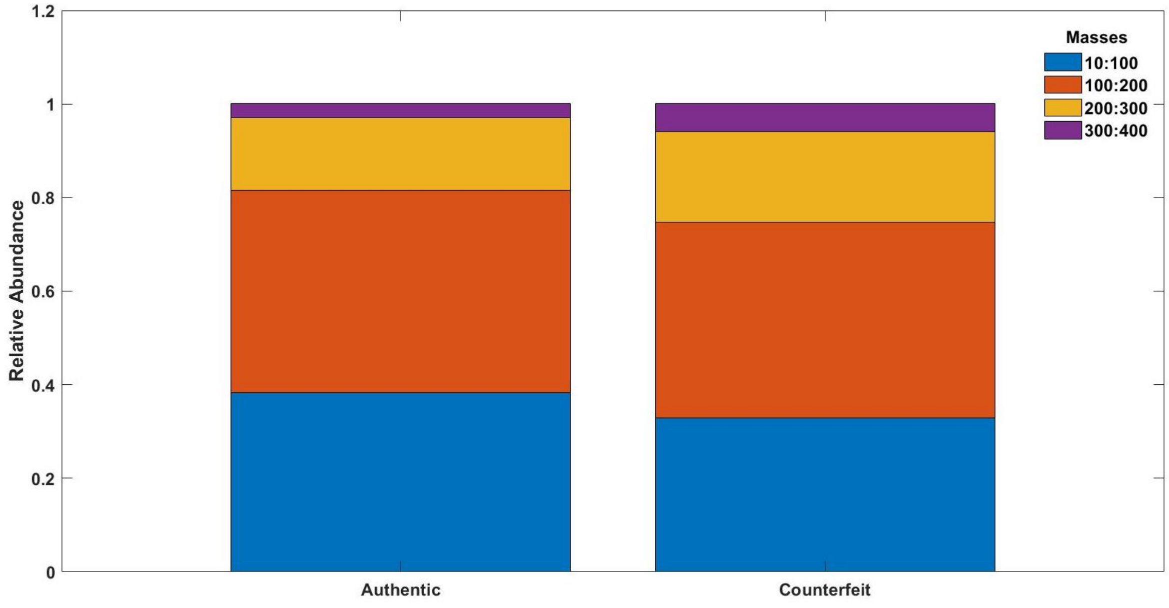

Figure S3: *Total ion current distribution defined over a set of m/z ranges (10:100, 100:200, 200:300 and 300:400)*

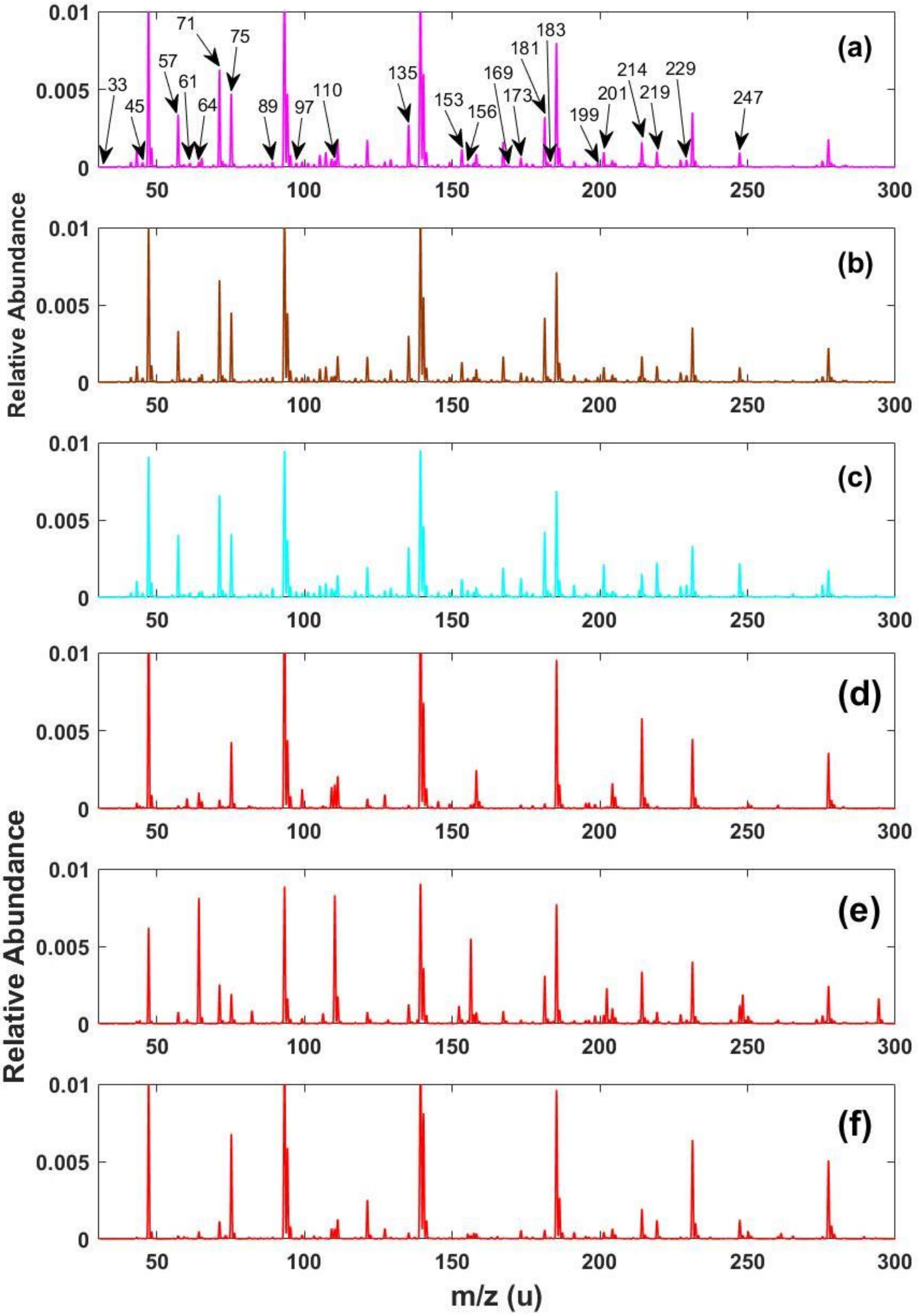

Figure S4: *DAPCI-MS spectral fingerprints from 3 authentic brands of whisky and 3 counterfeit samples with ethanol clusters remaining. (a) Mean spectra of Brand 1. (b) Mean spectra of Brand 2. (c) Mean spectra of Brand 3. (d) Individual counterfeit sample S16-0226. (e) Individual counterfeit sample S16-0810. (f) Individual counterfeit sample S16-0940.*

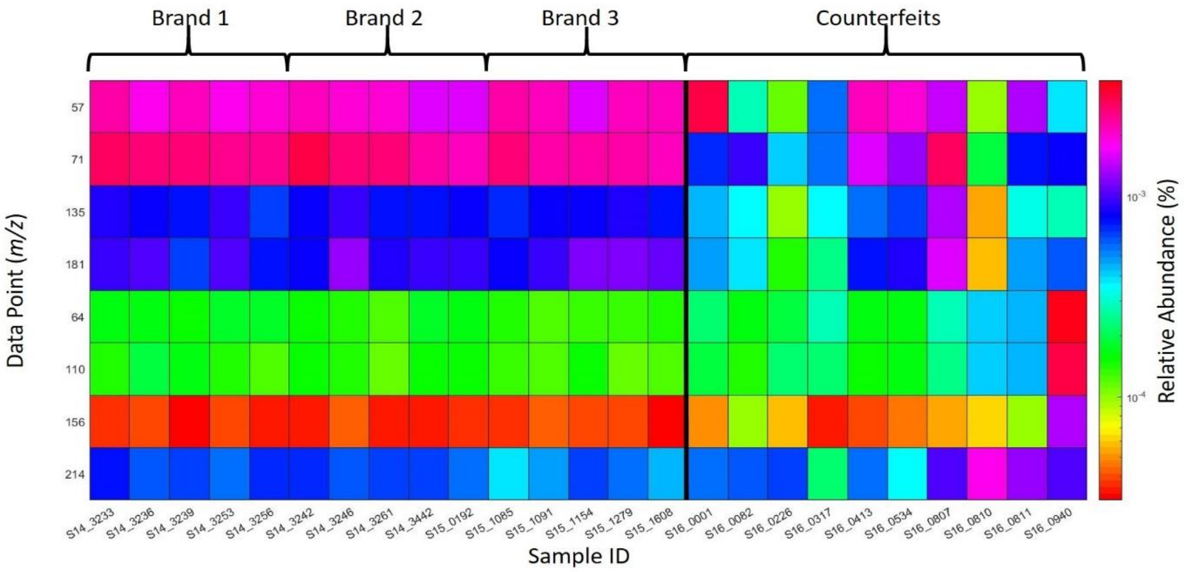

Figure S5*: Relative Intensity heat map for the authenticity markers identified by PCA analysis.*

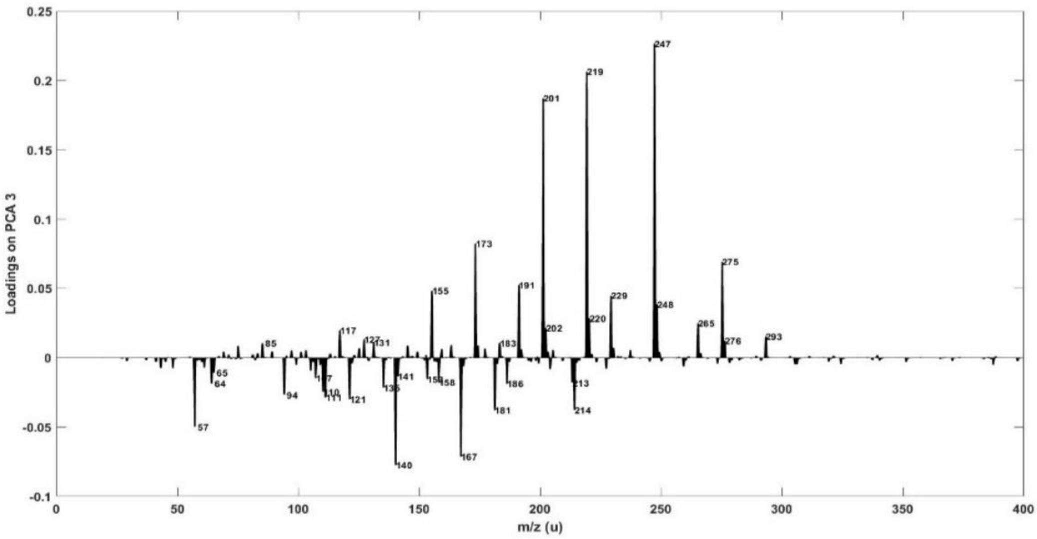

Figure S6*: Principal component loading results for principal component 3.*

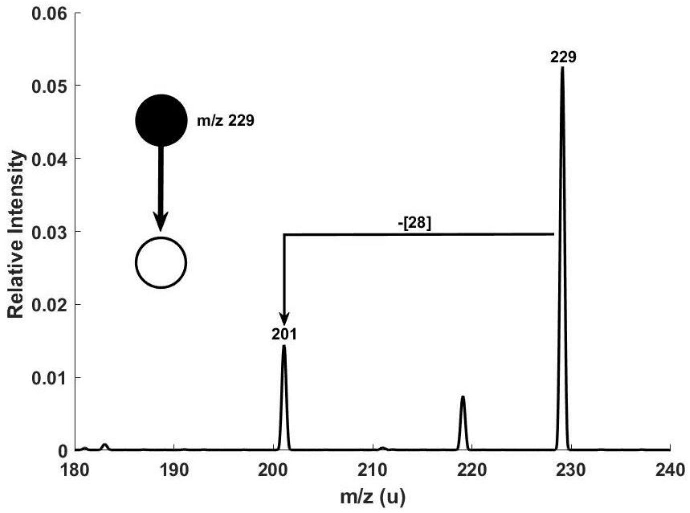

***Figure S7**: MS-MS data for CID of m/z 229 (suspected Ethyl Dodecanoate)*

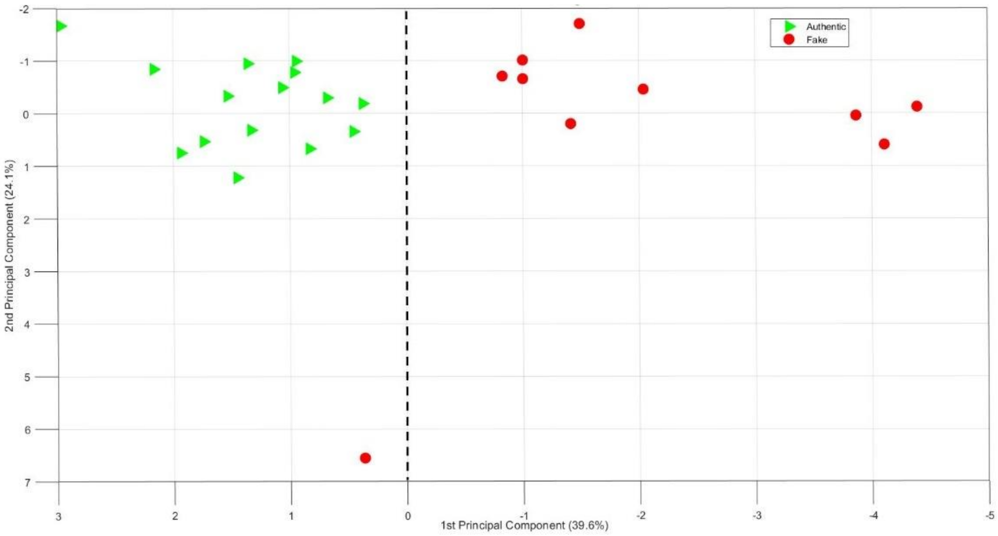

***Figure S8:** Principal component analysis from DAPCI-MS interrogation of Scotch using only a small selection of variables.*

Row Number	Sample Number	Sample Description
1	S14-3233	Blended Scotch Whisky Brand 1
2	S14-3236	Blended Scotch Whisky Brand 1
3	S14-3239	Blended Scotch Whisky Brand 1
4	S14-3253	Blended Scotch Whisky Brand 1
5	S14-3256	Blended Scotch Whisky Brand 1
6	S14-3242	Blended Scotch Whisky Brand 2
7	S14-3246	Blended Scotch Whisky Brand 2
8	S14-3261	Blended Scotch Whisky Brand 2
9	S14-3442	Blended Scotch Whisky Brand 2
10	S15-0192	Blended Scotch Whisky Brand 2
11	S15-1085	12 Year old Blended Scotch Whisky Brand 3
12	S15-1091	12 Year old Blended Scotch Whisky Brand 3
13	S15-1154	12 Year old Blended Scotch Whisky Brand 3
14	S15-1279	12 Year old Blended Scotch Whisky Brand 3
15	S15-1608	12 Year old Blended Scotch Whisky Brand 3
16	S16-0001	Known Fake 1
17	S16-0082	Known Fake 2
18	S16-0226	Known Fake 3
19	S16-0317	Known Fake 4
20	S16-0413	Known Fake 5
21	S16-0534	Known Fake 6
22	S16-0807	Known Fake 7
23	S16-0810	Known Fake 8
24	S16-0811	Known Fake 9
25	S16-0940	Known Fake 10

***Table S1**: List of samples used in this study*
